# Hospitals and Opportunities

**Published:** 1904-07-16

**Authors:** 


### Hospitals and Opportunities.

It is frequently urged, and the proposition is beyond dispute, that the medical profession shares an undue proportion of the burden of charitable service in the shape of the time, labour, and skill given by the members of the honorary staffs of the several departments of our public hospitals. Calculated on a commercial basis, these factors would in all truth represent an annual charge of no mean amount. They involve much unprofitable labour to those who thus render gratuitous service, and they also carry incalculable benefits to the sick and injured poor. Of recent years it has been recognised, at least to some extent, that the benefits resulting from the existing hospital system are by no means restricted to those who are in immediate attendance as patients. The gratuitous attendances of the staff and the clinical instruction offered to students and junior practitioners mean opportunities for improved medical knowledge and skill, the influence of which reaches all classes. And here, again, it must be remembered that much of this clinical instruction is undertaken by members of the staff without either fee or reward. Hospital physicians and surgeons may therefore present a striking record of time and effort spent gratuitously in public service and in the cause of charitable assistance and relief. There need not be any attempt to emphasise this statement, for no one at all acquainted with the facts will be disposed to contradict or even to qualify it. Not that medical men in general, or hospital staffs in particular, are, or pretend to be, more philanthropic than their neighbours. But their opportunities in this direction are more numerous, and, with rare exceptions, they do not avoid the claim. There is, however, another aspect of the question of hospital service which ought to be borne in mind. It is true the medical staff of a hospital gives freely, and that of its best, to the hospital and to the poor who form the patients. But it cannot be denied that some contribution is made in the opposite direction. A position on the staff means dignity and distinction and, in a greater or less measure, special experience. These elements, all will admit, carry weight with the public and have no insignificant influence on the attainment of success. It would be an idle task to attempt to strike the balance between what is given and what is gained. All that is necessary for our immediate purpose is to secure a recognition of the proposition that whilst hospital service means much gratuitous and thankless labour it has, even in the world of practical success, some measure of compensation. But yet, again, it may be reasonably contended that the duty of a member of a hospital staff is not limited to the mere treatment of patients who come under his immediate care. He enjoys large professional opportunities, and therefore to a corresponding extent responsibilities are required at his hand. In so far as fortune has given to him chances above his fellows, he is debtor to his profession and to his art in proportionate measure. In all justice and fairness he may be called upon to give an account of his stewardship and to show that he has used wisely and to good purpose the opportunities attached to his position. Possibly this cannot be done in the form of a formal inquisition, but the somewhat vague, though not unreal, tribunal of general professional opinion will certainly not fail to record a verdict. The repute of a hospital and its staff is without question judged by the amount and quality of the work they produce. It is a fair test and a reasonable demand. The criticism is perchance well-founded that the present generation suffers from a glut of medical, as of other classes of literature. But this in substance if not in form refers to quality rather than quantity. In any event, it is proper to ask the question whether the enormous clinical material of our hospitals is as fully utilised as it ought to be for the advance of medical science and the instruction of the general body of the profession. Upon every hospital staff there press many responsibilities, and not the lightest of these is the duty of adding to the region of exact knowledge contributions commensurate with the opportunities fate and fortune have placed in their hands.

